# Constitutive androstane receptor (CAR) functions as a tumor suppressor via regulating stemness in liver cancer

**DOI:** 10.1038/s41598-024-81571-z

**Published:** 2024-12-28

**Authors:** Sarah Da Won Bae, Romario Nguyen, Lawrence Yuen, Vincent Lam, Jacob George, Liang Qiao

**Affiliations:** 1 Storr Liver Centre, Westmead Institute for Medical Research, Department of Medicine, the University of Sydney at Westmead Hospital, Westmead, NSW 2145 Australia; 2 Department of Surgery, Westmead Hospital, Westmead, NSW 2145 Australia; 3 School of Medicine, University of Sydney, Sydney, Australia

**Keywords:** CITCO, Constitutive androstane receptor (CAR), Hepatocellular carcinoma (HCC), Liver cancer, Liver cancer stem cells (LCSCs), Tumor suppressor, Cancer, Cancer genetics, Cancer genomics, Cancer stem cells, Gastrointestinal cancer

## Abstract

Constitutive androstane receptor (CAR) is a xenosensor that is almost exclusively expressed in the liver. Studies in rodents suggest an oncogenic role for CAR in liver cancer, but its role in human liver cancer is unclear. We aimed to investigate the functional roles of CAR in human liver cancer with a focus on the liver cancer stem cells. We used bioinformatics to increase our understanding of CAR in human liver cancer and associated stem cell markers. We studied the functional roles of CAR in human liver cancer with a focus on the liver cancer stem cell using siRNA, modulation of CAR activity, and tumorsphere formation assays. We have revealed significant associations between CAR and a wide variety of signalling pathways including stemness signalling. Further in vitro studies have shown that activation of CAR significantly reduces cancer cell stemness and represses proliferation, migration, invasion, and the tumorsphere-forming abilities of liver cancer cells (p < 0.05). Our data demonstrates the unequivocal tumor-suppressive role of CAR in liver cancer. While more detailed mechanistic studies are warranted, the efficacy of CAR xeno-activators in the treatment of advanced hepatocellular carcinoma (HCC) may potentially open a new avenue for liver cancer therapy.

## Introduction

Constitutive androstane receptor (CAR) is a nuclear receptor^1^ encoded by the gene *NR1I3* (nuclear receptor subfamily 1 group I member 3)^2^. In humans, CAR is almost exclusively expressed in the liver (Human Protein Atlas available at: http://www.proteinatlas.org) with trace amounts detected in the duodenum, brain, heart, and kidney^3^. CAR is known for its role as a xenosensor but also plays important roles in normal liver physiology and liver regeneration, and in drug and energy metabolism, by regulating the transcription of target genes^4^.

The role of CAR in tumorigenesis is controversial. As summarized by us^5^, activation of CAR in mice models enhances aberrant cell proliferation and facilitates hepatocarcinogenesis through activation of multiple pathways such as gadd45b, anti-apoptosis, β-catenin, c-Myc and Cytochrome P450 2B6 (*CYP2B*)^6–9^. However, activation of CAR in a human background does not exert the same tumorigenic effects as in animal models^10–12^. Thus, it has been proposed that the biological functions and mechanisms of action of CAR in the pathogenesis of liver cancer in mice may not apply to humans^13,14^. Hepatocellular carcinoma (HCC) is an aggressive cancer comprising a heterogeneous population of cancer cells including mature cancer cells and liver cancer stem cells (LCSCs), the latter represents a subset of cells with strong self-renewing ability, differentiation potential, and enhanced refractoriness to drug-induced killing^15^.

Recent studies suggest that CAR may be a tumor suppressor in human liver and brain cancers. For instance, activation of CAR by CITCO (6-(4-Chlorophenyl)imidazo[2,1-b][1,3]thiazole-5-carbaldehyde-O-(3,4-dichloro benzyl)oxime), a human CAR-specific agonist, inhibited the growth and expansion of brain tumor stem cells^16^. Our recent studies have revealed that CAR is a tumor suppressor and a potential biomarker for favourable prognosis of liver cancer^17^. In a study on liver cancer using Hep3B and HepG2 cells with the tet-on system and verified by soft agar colony assays and xenograft models, Li et al. observed that CAR represses hepatocarcinogenesis by inhibiting erythropoietin signalling^18^. This study however did not investigate the role of xenobiotic-induced CAR activation, as has been done in rodents^18^. Due to the species differences, such studies can only be performed in human cancer cells in vitro^5,19–21^.

Liver cancer, mainly in the form of hepatocellular carcinoma (HCC), is well known for its inter- and intra-tumoral heterogeneity and malignancy^22^. The aggressiveness of HCC can be attributed to the presence of liver cancer stem cells (LCSCs)^23^. LCSCs only represent a small portion of liver cancer cells and can be identified by stemness markers such as CD24, CD44, CD133 and EpCAM^24^. Despite of small numbers, LCSCs are known to be heavily involved in HCC recurrence and drug resistance. As such, LCSCs are recognized as an ideal therapeutic target for liver cancer therapy. We hypothesized that CAR acts as a tumor suppressor by regulating the stemness of liver cancer cells. We first undertook bioinformatics analyses of human HCC to explore the clinical relevance of CAR and then conducted a series of functional assays in multiple HCC cell lines and derived LCSCs to validate the tumor suppressor role of CAR in human HCC.

## Materials and methods

### Bioinformatics analysis of CAR expression and clinical implications

Gene sets related to cancer prognosis, survival, recurrence, progression, proliferation, invasion, metastasis, angiogenesis, tumor immune response, stem cell features, signalling pathways involved in cancer growth and survival, as well as HCC specific gene sets were downloaded from Molecular Signatures Database V7.0 (MSigDB) and cBio cancer genomics portal (cBioPortal)^25^. Gene signatures that were obtained from cBioPortal have been given the prefix “cBioPortal” and those obtained from MsigDB were assigned the studies’ author as the prefix. The top 20 genes associated with favourable and unfavourable survival, total of 2 gene sets for liver cancer were obtained from Protein Atlas and were given the prefix “Protein Atlas”. Spearman correlation between NR1I3 expression and gene sets related to HCC were analysed using Gene Expression Profiling Interactive Analysis 2 (GEPIA2)^26^. Spearman correlation coefficients and their respective *p* values were collated into Supplementary Table 1. Negatively and positively correlated gene sets were separated into two groups and within each group, the gene sets that met the inclusion criteria were selected. In this study, Spearman’s correlation of − 0.5 and 0.5 represents a moderate negative and positive correlation, respectively. Any gene sets that did not have a moderate correlation to CAR were omitted. A *p* ≤ 0.05 is considered statistically significant.

Data on the expression of NR1I3 and stem cell markers in liver cancer samples were downloaded from cBioPortal, GSE14520, GSE36376, GSE63898, GSE76297, GSE5975, GSE63898, GSE76297, GSE5975, GSE20238, GSE1898 and GSE76427 (Supplementary Table 2). Microarray data for GDC TCGA Liver Hepatocellular Carcinoma (LIHC) was downloaded from MEXPRESS^27^. Correlation between NR1I3 and stem cell markers was assessed using GEPIA2 and presented in a heat map. Spearman correlation coefficients and their respective *p* values were collated into Supplementary Table 3.

### Cell culture

Three human HCC cell lines, Hep3B (Cat. No. 86062703, Lot No. 18I014), Huh-7 (Cat. No. JCRB0403, Lot. No. 08282017), and PLC/PRF/5 (Cat. No. 85061113, Lot No. 10D004) were obtained from Cell Bank Australia. Hep3B and PLC/PRF/5 were cultured in Dulbecco’s Modified Eagle Medium (Lonza, Basel, Switzerland) supplemented with 10% fetal bovine serum (FBS) and Huh-7 was cultured in Dulbecco’s Modified Eagle Medium with low glucose (Sigma-Aldrich, St. Louis, Missouri, USA) supplemented with 10% FBS. The human hepatocyte cell line, IHH, was cultured in Dulbecco’s Modified Eagle Medium (Lonza) supplemented with 10% FBS. STR profiling has been performed for all cell lines and mycoplasma contamination testing has been conducted (mycoplasma negative).

### Activation of CAR in HCC cells

HCC cells were treated with the human CAR agonist CITCO (Tocris Biosciences. Bristol, UK). Briefly, cells were exposed to 1 mM of CITCO in growth media for the indicated time, and dimethylsulfoxide was used as vehicle control. The effect of CITCO on CAR activation was confirmed by quantifying the gene expression of CAR-specific downstream targets including *CYP2A6, CYP2B6,* and *UGT1A1* by RT-qPCR (Suppl. Fig. 1).

### Modulation of CAR expression in HCC cells

CAR expression in HCC cells was downregulated by using CAR-specific siRNAs or overexpressed by using a plasmid carrying human CAR encoding NR1I3. Based on a series of titration assays in three HCC cell lines, the optimal concentration of CAR-specific siRNAs (siCAR) (Dharmacon, Lafayette, Colorado, USA) was determined to be 40 nM at which concentration, > 60% knockdown of CAR expression was achieved (Suppl. Fig. 2) and thus this concentration was used in subsequent studies. For knockdown studies, cells were seeded onto 6-well plates and grown to 70% confluency. Cells were then transfected with siCAR for 48 h before functional assays as described below.

To overexpress CAR, HCC cells were transfected with the expression plasmid containing human CAR encoding gene NR1I3 (pCMV6-Entry-NR1I3) (Origene Technologies, Rockville, Maryland, USA) in Opti-MEM reduced-serum containing FuGene (Promega, Madison, Wisconsin, USA). Cells were transfected for 48 h before functional assays. Successful knockdown and overexpression of CAR were confirmed by RT-qPCR and western blot.

### Quantitative reverse transcription polymerase chain reaction (RT-qPCR)

Total RNA was extracted using FavorPrep Tissue Total RNA Kit (Favorgen Biotech Corporation, Taiwan) according to the manufacturer’s protocol and was used to generate cDNA using M-MLV reverse transcriptase (Promega). cDNA was diluted (1:10) for RT- RT-qPCR with QuantiNova SYBR Green PCR Kit (Qiagen, Hilden, Germany). RT-qPCR cycling conditions included an initial activation step (2 min, 95 °C), denaturation (5 s, 95 °C), and combined annealing/extension (10 s, 60 °C) for 35–40 cycles. RT-qPCR was performed using the CFX96 Touch Real-Time PCR Detection system (Bio-Rad, Hercules, California, USA). All qPCR data were analysed using the double delta Ct analysis. Primer sequences are listed in Supplementary Table 4.

### Western blot

The expression of CAR was examined by Western blot. In brief, total proteins from treated cells were extracted, protein concentration determined, and resolved on SDS–polyacrylamide gels (8%) and transferred onto polyvinylidene difluoride membranes. Membranes were blocked using 5% skim milk/TBST solution (tris-buffered saline, 0.1% Tween 20) for 1 h, followed by incubation with primary antibodies at 4 °C. Then membranes were incubated with horseradish peroxidase secondary antibodies for 1 h. Blots were developed using the West Pico and Femto chemiluminescent substrates (Thermo Fisher Scientific, Waltham, Massachusetts, USA). Protein bands were quantified with Image J (National Institutes of Health, Maryland, USA). All antibodies and dilutions used in this study are listed in Supplementary Table 5.

### Proliferation assay

Treated cells were seeded into 96-well plates (3000 cells/well) and maintained for 48 h. Cell Counting Kit-8 (CCK-8; Sigma-Aldrich) was used to measure the proliferation rate by optical density (OD)/absorbance using the SpectraMax iD5 Microplate Reader (Molecular Devices, San Jose, California, USA) at 450 nm.

### Transwell migration and invasion assay

Transwell cell culture inserts (diameter 6.5 mm, pore size 9 mm; Corning, New York, United States) were placed into wells with (for migration study) or without (for invasion study) Matrigel (200 mg/mL; Corning). Media supplemented with 10% FBS was added to the bottom of the well before placing the inserts in the wells. Cells were resuspended in their respective media without FBS and seeded into the inserts and incubated for 24 h (migration) and 48 h (invasion) in a 5% CO_2_ incubator at 37°C. Inserts were washed with PBS and then fixed with ice-cold methanol for 15 min. Inserts were stained with 1% crystal violet (Sigma-Aldrich) for 25 min and washed with distilled water. Cells were visualized using the Olympus CKX41 microscope and 5 representative fields were photographed at × 4 magnification for analysis. Images were analysed with Image J and the total area occupied was quantified using the colour threshold tool.

### Tumorsphere formation assay

Cells were cultured in ultra-low attachment plates (Sigma-Aldrich) in a 1:1 mixture of Dulbecco’s Modified Eagle Medium: Nutrient Mixture F12 supplemented with 100 IU/ml penicillin, 100 mg/ml streptomycin 20 ng/ml human epidermal growth factor recombinant protein, human fibroblast growth factor-basic recombinant protein, 2% B27 supplement minus vitamin A and 1% N-2 supplement. Tumorspheres were cultured for 4 weeks with the medium changed every 3–4 days in the presence or absence of CITCO. Tumorspheres were stained with Hoechst 33342 and visualized with ChemiDoc MP (Bio-Rad). Images were further analysed with Image J. Tumorspheres were collected for RNA extraction and cDNA was synthesized for subsequent RT-qPCR analysis.

### Cell cycle analysis

Cells were fixed with 70% ethanol at 4°C for 1 h, washed with phosphate-buffered saline solution (PBS, 2mM Ethylenediaminetetraacetic acid and 1% FBS), stained with DAPI (0.1 µg/ml) and analysed with BD LSR Fortessa Cell Analyser (Becton Dickinson, Franklin Lanes, New Jersey, USA). Data were analysed using the Cell Cycle Analysis tool on FlowJo™ v10.8 Software (Becton Dickinson). The percentage of cells in each phase (G1, S, and G2) were recorded and compared to the respective control groups.

### Statistical analysis

All statistical analyses were performed using GraphPad Prism version 8.0.0 for Windows (GraphPad Software, Boston, Massachusetts, USA). Differences in mRNA expression between any two groups were analysed by unpaired two-tailed *t*-test or paired *t*-test as appropriate. Ordinary one-way ANOVA tests followed by Tukey’s multiple comparisons test were used to test the differences among the multiple independent groups. For survival data analysis, the Kaplan–Meier curves were analysed by log-rank test. Correlations were assessed using Spearman correlation coefficients. Differences in proliferation, migration, invasion, sphere formation, and stem cell markers between control and either CITCO-treated or siCAR groups were analysed by unpaired two-tailed *t*-test, and data are represented as mean ± standard error of mean (SEM). A *p* ≤ 0.05 is considered statistically significant in all analyses.

## Results

### CAR expression correlates with genes involved in cancer development and drug resistance

**Fig. 1:**
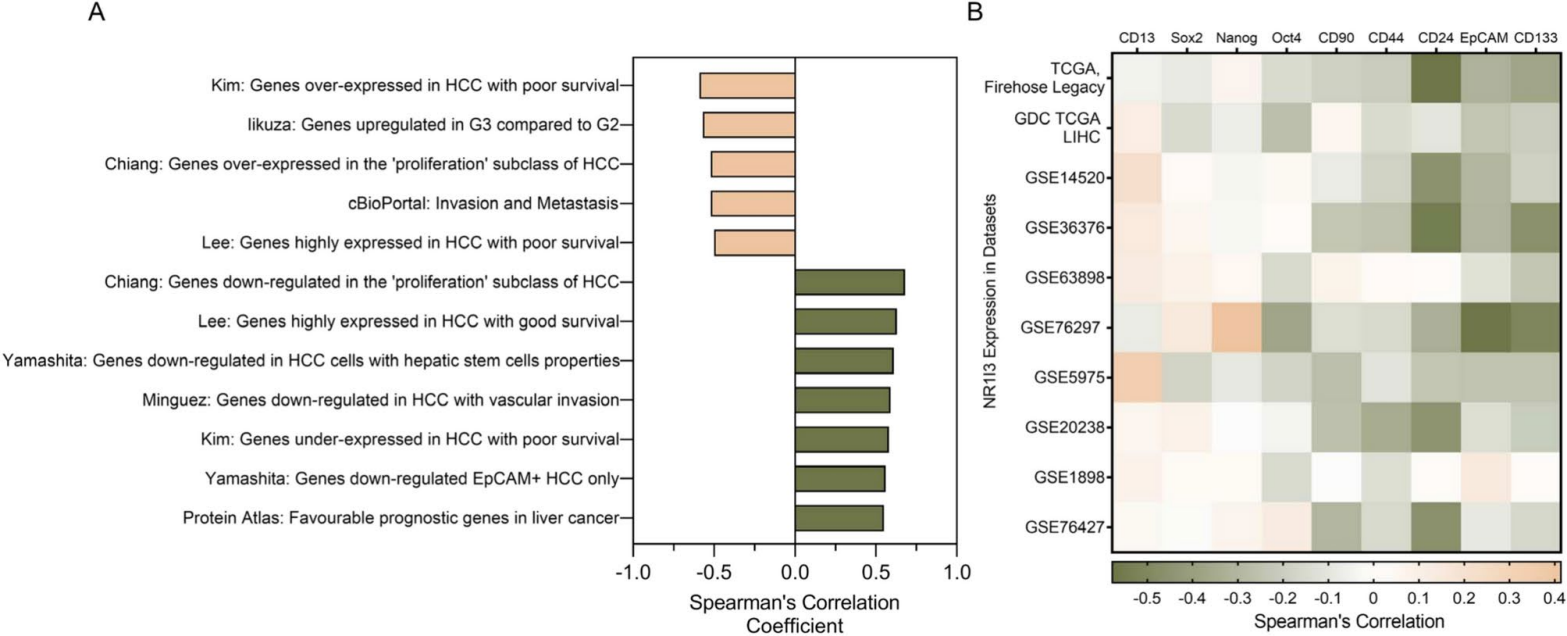
Correlation between CAR expression, gene signatures, and stem cell markers. Gene signatures were downloaded from MSigDB, the Human Protein Atlas, and cBioPortal. Spearman’s correlation analysis was performed using GEPIA2. CAR negatively correlated to genes that are over-expressed or involved in HCC growth and progression while the opposite trend was discovered with the gene signatures that are downregulated in high-grade HCC and those associated with better HCC survival (*p* < 0.05) (**A**). A significant negative correlation between CAR and *CD24*, *CD44*, *CD133,* and *EpCAM* can also be seen in the heatmap (*p* < 0.05) (**B**).

The correlation between CAR expression level in HCC with that of the genes involved in invasion, metastasis, cancer stem cell phenotype, HCC progression, and disease prognosis was assessed. As shown in Fig. 1A, CAR negatively correlated with the genes involved in invasion and metastasis as defined by cBioPortal (r =  − 0.52, *p* < 0.0001). A moderate positive correlation was seen between CAR expression and vascular invasion-related genes (Minguez: genes downregulated in HCC with vascular invasion, r = 0.59, *p* < 0.0001). CAR negatively correlated to genes over-expressed in the proliferative subclass of HCC (Chiang: genes over-expressed in the “proliferation” subclass of HCC, r =  − 0.52, *p* < 0.0001) but positively correlated with the genes that are down-regulated in the proliferative subclass of HCC (Chiang: genes down-regulated in the “proliferation” subclass of HCC, r = 0.68, *p* < 0.0001). A moderate negative correlation was observed between CAR expression and genes down-regulated during the transition from Grade 2 (G2) to Grade 3 (G3) (Iikuza: genes upregulated in G3 compared to G2, r =  − 0.57, *p* < 0.0001).

In HCC patients with poor survival, CAR expression negatively correlated with the over-expressed genes (Kim: genes over-expressed in HCC with poor survival, r =  − 0.59, *p* < 0.0001) whereas it positively correlated with the down-regulated genes (Kim: genes under-expressed in HCC with poor survival, r = 0.58, *p* < 0.0001). In HCC patients with poor survival, CAR expression negatively correlated with the over-expressed genes (Lee: genes highly expressed in HCC with poor survival, r =  − 0.5, *p* < 0.0001) whereas in the HCC patients with good survival, CAR expression positively correlated with the over-expressed genes (Lee: genes highly expressed in HCC with good survival, r = 0.63, *p* < 0.0001). CAR expression also positively correlated with the favourable prognostic genes from The Human Protein Atlas (r = 0.55, *p* < 0.0001).

### CAR expression correlates with stem cell markers in HCC

A moderate positive correlation was seen between CAR expression and genes down-regulated in Epithelial cell adhesion molecule (*EpCAM*)^+^ HCC (Yamashita: genes down-regulated *EpCAM*^+^ HCC only, r = 0.56, *p* < 0.0001) and genes down-regulated in HCC cells that are related to hepatic stem cell properties (Yamashita: genes down-regulated in HCC cells with hepatic stem cell properties, r = 0.61, *p* < 0.0001) (Fig. 1A).

The association between CAR expression and the expression pattern of stemness markers in HCC samples was analysed by Spearman’s Correlation analysis. A general trend of negative association between CAR and the most reported stemness markers in HCC (Cluster of differentiation 24 (*CD24*), *CD44*, Prominin-1; *CD133*, *EpCAM*) was identified (Fig. 1B). More specifically, there is a moderate negative correlation between CAR and *CD24* in 50% (5 out of 10) of the datasets (r < − 0.5, *p* < 0.05). CAR expression in the TCGA, Firehose Legacy shows the strongest negative association with *CD24* compared to all other datasets (r =  − 0.58, *p* < 0.05). In GSE76297, CAR has a moderate negative correlation with *EpCAM* and *CD133* (r =  − 0.58, − 0.51, respectively, *p* < 0.05). Weaker negative associations between CAR expression and other stemness markers were seen across all datasets although not statistically significant (Suppl. Table 3).

### Functional roles of CAR in HCC cells

#### CAR activation hinders the proliferation, migration, invasion, and sphere-forming ability of HCC cells

**Fig. 2:**
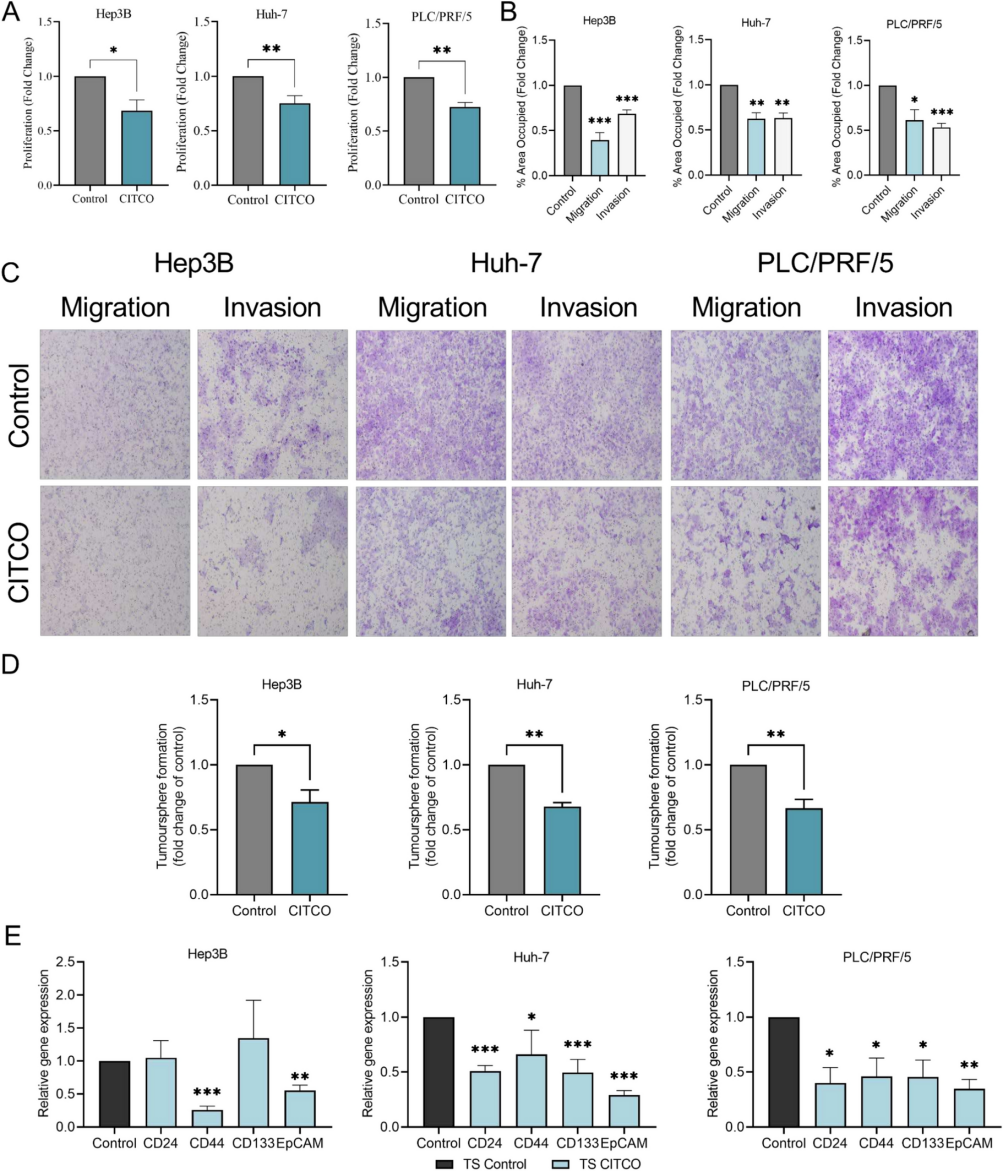
CAR activation decreased the proliferation, migration, invasion, and tumorsphere formation of HCC cells. HCC cells were pre-treated with 1 µM of CITCO for 48 h, and then further cultured in the presence of 1 µM of CITCO for 24 h (for migration assay) or 48 h (for proliferation and invasion assay). A significant decrease in proliferation (**A**), migration, and invasion (**B**) was seen in Hep3B, Huh-7, and PLC/PRF/5 cells. (**C**) Representative images of migration and invasion of HCC cells treated with CITCO. (**D**) Quantitative analysis of the tumorsphere formation assay showing decreased sphere-forming ability in CITCO-treated cells. (**E**) Significant reductions in the gene expression of stem cell markers in CITCO-treated tumorspheres. *p < 0.05; **p < 0.01; ***p < 0.001. n = 3 per group. n = 3 per group. Data are represented as mean ± SEM.

To investigate the biological functions of CAR in the pathogenesis of HCC, we activated CAR in HCC cells using CITCO, a human CAR-specific agonist. The effect of CAR activation on cell proliferation was assessed by CCK-8. Activation of CAR significantly decreased the proliferation (Fig. 2A), migration, and invasion (Fig. 2B,C) of all three cell lines (Fig. 2B,C). CITCO treatment also decreased tumorsphere formation consistently across three cell lines (Fig. 2D). Then, RNA was extracted from these tumorspheres and the expression of major stem cell markers was studied. CITCO treatment decreased the expression of all stem cell markers in the tumorspheres derived from Huh-7 and PLC/PRF/5 cells (Fig. 2E), while only *CD44* and *EpCAM* expression was significantly reduced in the CITCO treated tumorspheres derived from Hep3B cells (Fig. 2E). Meanwhile, an acute increase in the percentage of cells in the S phase following CITCO treatment together with a concomitant decrease in the percentage of cells in the G1 phase was observed (Suppl. Fig. 3), although these changes did not reach statistical significance.

#### Over-expression of CAR does not affect cell proliferation, migration, or invasion

To clarify the roles of CAR in HCC, we used a CAR over-expressing plasmid. Over-expression of CAR reduced the proliferation of Huh-7 cells (*p* < 0.05) but not the other two cell lines (Suppl. Fig. 4A). Meanwhile, CAR over-expression did not alter the migration and invasion of all three cell lines (Suppl. Fig. 4B,C).

#### CAR plays an important role in the proliferation of HCC cells

**Fig. 3:**
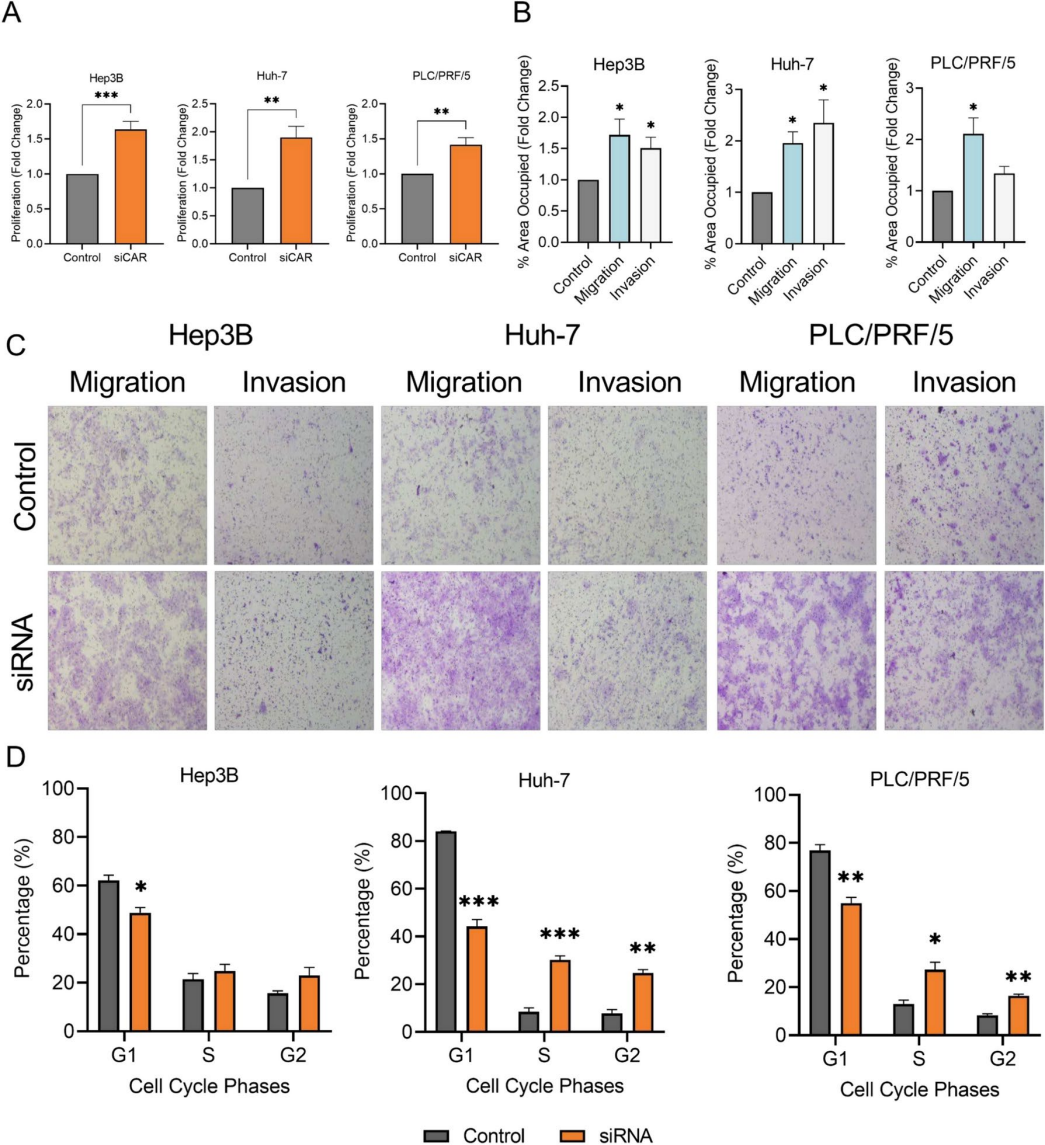
Effects of CAR knockdown on the proliferation, migration, invasion, and cell cycle distribution of HCC cells. Transient CAR knockdown by siCAR led to a significant increase in the proliferation of HCC cells (**A**). CAR knockdown also enhanced migration and invasion across all three cell lines (**B**). (**C**) Representative images of migration and invasion of HCC cells with CAR knockdown. (**D**) CAR knockdown resulted in a decreased proportion of cells in the G1 phase and a concomitant increase in the proportion of cells in the S and G2 phases. *p < 0.05; **p < 0.01; ***p < 0.001. n = 3 per group. Data are represented as mean ± SEM.

To further elucidate the biological roles of CAR in HCC, CAR expression was knocked down using CAR siRNA (siCAR). As shown in Fig. 3A, the knockdown of CAR significantly increased the proliferation of Hep3B (by 1.6-fold), Huh-7 (by 1.9-fold), and PLC/PRF/5 (by 1.4-fold) cells (*p* < 0.01). As shown in Fig. 3B,C, knockdown of CAR also significantly increased the migration of Hep3B (by 1.7-fold), Huh-7 (by twofold), and PLC/PRF/5 (by 2.1-fold) cells. Additionally, CAR knockdown resulted in a significant increase in the invasion of Hep3B and Huh-7 cells, and a similar trend was observed in PLC/PRF/5 cells.

We examined the effect of CAR knockdown on cell cycle progression. CAR knockdown significantly decreased the proportion of cells in the G1 phase of Hep3B (by 14%), Huh-7 cells (by 40%), and PLC/PRF/5 (by 22%) cells, with a concomitant increase in the percentage of cells seen in S and G2 phases in Huh-7 (by 22% and 17%, respectively) and PLC/PRF/5 cells (by 14% and 8%, respectively) (Fig. 3D, p < 0.05). A trend of increase in the percentage of cells in the S and G2 phases was observed in Hep3B cells.

#### CAR regulates the expression of stem cell markers

To study the impact of CAR on the expression of liver cancer stem cells, we modulated CAR by activation (using CITCO, in HCC cells and tumorspheres derived from HCC cells) and by CAR knockdown (using siCAR, in HCC cells). Based on bioinformatics analyses (Fig. 1B), CAR is significantly negatively correlated with the expression level of several key markers of cancer stem cells including *CD24, CD44, CD133,* and *EpCAM*. Thus, we verified the regulatory effect of CAR on stemness features. CAR activation by CITCO significantly decreased *CD24* expression in Hep3B and PLC/PRF/5 cells with a decreasing trend seen in Huh-7 cells (Suppl. Fig. 5A). The impact of CAR activation on the expression of these stem cell markers was even more prominent in the tumorspheres, where CITCO significantly reduced the expression of multiple cancer stem cell markers including CD44 and EpCAM (in the tumorspheres derived from all three cell lines), *CD24, CD44, CD133* and *EpCAM* (in the tumorspheres derived from Huh-7 and PLC/PRF/5 cells) (Fig. 2E). Knockdown of CAR resulted in increased expression of the above four cancer stem cell markers in all three HCC cell lines although only significant for *CD133* in PLC/PRF/5 cells (Suppl. Fig. 5B).

## Discussion

CAR is almost exclusively expressed in the liver. Thus, deciphering its precise function could potentially lead to the discovery of a liver-specific target for the therapy of liver disease. For the first time we have studied the association between CAR and various cellular pathways. Of note, CAR negatively correlated to genes that are over-expressed or involved in HCC growth and progression while the opposite trend was discovered with the gene signatures that are downregulated in high-grade HCC and those associated with better HCC survival. These associations give us an insight into the possible pathways by which CAR acts as a tumor suppressor. Using bioinformatic analyses we show that CAR expression negatively correlated with the expression of the stem cell markers *CD24, CD44, CD133,* and *EpCAM*, which were also decreased following CITCO treatment. In functional assays, CAR activation reduced cancer cell proliferation, migration, and invasion; these effects were reversed by siRNA-mediated CAR knockdown. Finally, CAR activation impacted stemness features of HCC cells leading to reduced expression of stem cell markers and impaired the ability to form tumorspheres.

Cancer stem cells are identified using cell surface markers and although no markers are exclusively specific to LCSCs^15^, we identified *CD24, CD44, CD133,* and *EpCAM* that are correlated to CAR expression. A novel finding is that CAR activity impacts the functionality of liver cancer stem cells. CAR activation (by CITCO) decreased the stemness of these cells with decreased expression of *CD24* and *CD133*, and this was reversed by siCAR. Notably, the effects on stemness features were recapitulated in tumorspheres derived from these cells following CAR activation. Our results support the hypothesis that CAR acts as a tumor suppressor by its impacts on LCSCs. It is established that *CD24* and *CD133* are involved in propagating uncontrolled proliferation, migration, and invasion in HCC^28–31^. Therefore, as expected, repression or downregulation of these markers by CAR activation, attenuated cancer growth and migration. Consistently, a previous study found that CITCO activation of CAR resulted in a decrease in *CD133*^+^ brain tumor stem cells^16^.

Uncontrolled cell proliferation as well as increased migratory and invasive capabilities are key features of malignant cells. In rodent models, CAR stimulates cell proliferation and promotes liver regeneration and thus promotes cancer formation^32–35^. In contrast, our study showed that activation of CAR resulted in a reduction in the proliferation, migration, and invasion of human HCC cells, consistent with a tumor-suppressive role. The inhibitory effects of CAR were reduced by siRNA-mediated CAR knockdown which resulted in a concomitant increase in the proportion of cells in the S and G2 phases of the cell cycle. These functional readouts are supported by our previous bioinformatics analyses where decreasing CAR expression was seen in more advanced and later-stage HCCs^17^. Interestingly, forced CAR overexpression, unlike that by Li et al.^18^, did not impact proliferation, migration, or invasion. This suggests that activation of CAR activity but not merely its overexpression is required for the tumor suppressive functions. Our findings support a need for further studies on the role of CAR activators as an approach to liver cancer therapy.

Whilst the species differences of CAR limit us to in vitro studies, our data may seem correlative and lacking in mechanistic data, however, our robust data demonstrate an unequivocal tumor-suppressive role of CAR in liver cancer. Our data opens a new pathway for developing more effective anticancer therapy for liver cancer using CAR as a potential target. More detailed mechanistic studies as well as the efficacy of CAR xeno-activators in the treatment of advanced HCC warrants further study.

## Supplementary Information

Supplementary Information.

## Data Availability

All data are available upon request from the corresponding authors.

## Abbreviations

CAR: Constitutive androstane receptor
CCK-8: Cell counting kit-8
CITCO: 6-(4-Chlorophenyl)imidazo[2,1-b][1,3]thiazole-5-carbaldehyde-O-(3,4 dichlorobenzyl)oxime
FBS: Fetal bovine serum
GEO: Gene expression omnibus
GEPIA2: Gene Expression Profiling Interactive Analysis 2
HCC: Hepatocellular carcinoma
LCSCs: Liver cancer stem cells
LIHC: Liver hepatocellular carcinoma
MSigDB: Molecular Signatures Database V7.0
NR1I3: Nuclear receptor subfamily 1 group I member 3
RT-qPCR: Quantitative reverse transcription polymerase chain reaction
siCAR: Cells transfected with CAR specific siRNA
