# Supplementary material for: Constitutive androstane receptor (CAR) functions as a tumor suppressor via regulating stemness in liver cancer
Source: Sci Rep. 2024 Dec 28;14:30926. doi: 10.1038/s41598-024-81571-z (PMC11680565; doi:10.1038/s41598-024-81571-z)
Supplement: Supplementary file 1 — Supplementary Information. [file 41598_2024_81571_MOESM1_ESM.pdf]

Constitutive androstane receptor (CAR) functions as a tumor suppressor via regulating stemness in liver cancer

Sarah Da Won Bae[a], Romario Nguyen[a], Lawrence Yuen[b], Vincent Lam[b], Jacob George[a*], Liang Qiao [a*]

a Storr Liver Centre, Westmead Institute for Medical Research, University of Sydney, and Westmead Hospital, Westmead, NSW 2145, Australia.

b Department of Surgery, Westmead Hospital, Westmead, NSW 2145, Australia.

*Correspondence Author: Jacob George (jacob.george@sydney.edu.au) and Liang Qiao (liang.qiao@sydney.edu.au; https://orcid.org/0000-0002-4723-8935), Storr Liver Centre, Westmead Institute for Medical Research, University of Sydney, Westmead, NSW 2145, Australia. Phone: 612-86273534; Fax: 612-86273099.

Acknowledgments and funding: JG is supported by the Robert W. Storr Bequest to the Sydney Medical Foundation, University of Sydney; National Health and Medical Research Council of Australia (NHMRC) Program and Investigator Grants (AAP2008983, APP1053206, APP1196492). The study was supported by project grants from NSW Cancer Council (APP1145008 to JG and LQ; APP1070076 to CL and LQ), and Cancer Institute NSW grants 15/TRC/1-01 and 2021/ATRG2028, and a NHMRC Program Grant (APP1149976 to JG).

Contents

Supplementary Materials and Methods 3

S. Table 1. Correlation between NR1I3 and gene signatures by Spearman analysis 3

S. Table 2. Platforms and sample numbers used in this study 4

S. Table 3. Correlation between NR1I3 and liver cancer stem cell markers by Spearman's correlation 5

S. Table 4. Primers used in this study 6

S. Table 5. Reagents for Western blot used in this study 7

Supplementary Figures 7

S. Figure 1. Successful CAR activation in HCC cells. 7

S. Figure 2. Successful CAR knockdown in HCC cells. 8

S. Figure 3. Cell cycle analysis in HCC cells treated with CITCO 8

S. Figure 4. Effect of CAR over-expression on the proliferation, migration, and invasion of HCC cells. 9

S. Figure 5. Effects of CAR modulation on the expression of cancer stem cell markers. 10

Supplementary Materials and Methods

S. Table 1. Correlation between NR1I3 and gene signatures by Spearman analysis

Gene Signatures	Standard name (MSigDB)	PMID	Correlation coefficients	*p*
Kim: genes over-expressed in HCC with poor survival	KIM_LIVER_CANCER_POOR_SURVIVAL_UP	21320499	-0.59	2.90E-36
Iikuza: Genes upregulated in G3 compared to G2	IIZUKA_LIVER_CANCER_PROGRESSION_G2_G3_DN	15710396	-0.57	8.60E-33
Chiang: Genes over-expressed in the 'proliferation' subclass of HCC	CHIANG_LIVER_CANCER_SUBCLASS_PROLIFERATION_UP	18701503	-0.52	1.20E-26
cBioPortal: Invasion and Metastasis			-0.52	2.80E-27
Lee: Genes highly expressed in HCC with poor survival	LEE_LIVER_CANCER_SURVIVAL_DN	15349906	-0.5	1.90E-24
Chiang: Genes down-regulated in the 'proliferation' subclass of HCC	CHIANG_LIVER_CANCER_SUBCLASS_PROLIFERATION_DN	18701503	0.68	1.20E-51
Lee: Genes highly expressed in HCC with good survival	LEE_LIVER_CANCER_SURVIVAL_UP	15349906	0.63	1.90E-42
Yamashita: Genes down-regulated in HCC cells with hepatic stem cells properties	YAMASHITA_LIVER_CANCER_STEM_CELL_DN	19150350	0.61	2.90E-39
Minguez: Genes down-regulated in HCC with vascular invasion	MINGUEZ_LIVER_CANCER_VASCULAR_INVASION_DN	21320499	0.59	1.20E-35
Kim: Genes under-expressed in HCC with poor survival	KIM_LIVER_CANCER_POOR_SURVIVAL_DN	21320499	0.58	2.70E-34
Yamashita: Genes down-regulated in $EpCAM^+$ HCC only	YAMASHITA_LIVER_CANCER_WITH_EPCAM_DN	18316609	0.56	7.20E-32
Protein Atlas: Favourable prognostic genes in HCC			0.55	5.00E-31

S. Table 2. Platforms and sample numbers used in this study

GEO Accession	Platform	NT	T
GSE14520	GPL3921 (Affymetrix HT Human Genome U133A Array)	220	225
GSE22058	GPL6793 (Rosetta/Merck Human RSTA Custom Affymetrix 1.0 microarray)	97	97
GSE25097	GPL10687 (Rosetta/Merck Human RSTA Affymetrix 1.0 microarray, Custom CDF)	243	243
GSE36376	GPL10558 (Illumina HumanHT-12 V4.0 expression beadchip)	193	240
GSE57957	GPL10558 (Illumina HumanHT-12 V4.0 expression beadchip)	39	39
GSE57958	GPL8490 (Illumina Human Methylation 27 beadchip (HumanMethylation27_270596_v.1.2)	59	61
GSE60502	GPL96 ([HG-U133A] Affymetrix Human Genome U133A Array)	18	18
GEO (gene expression omnibus), HCC (hepatocellular carcinoma)			

NT: non-tumors; T: HCC tumors

S. Table 3. Correlation between NR1I3 and liver cancer stem cell markers by Spearman's correlation

	CD13	Sox2	Nanog	Oct4	CD90	CD44	CD24	EpCAM	CD133
TCGA, Firehose Legacy	-0.06 (p=0.5476)	-0.09 (p=0.3639)	0.07 (p=0.4944)	-0.15 (p=0.1290)	-0.19 **(p=0.0475)**	-0.22 **(p=0.0522)**	-0.58 **(p<0.0001)**	-0.33 **(p=0.0006)**	-0.38 **(p<0.0001)**
GDC TCGA LIHC	0.12 (p=0.1560)	-0.15 (p=0.0538)	-0.07 (p=0.3465)	-0.27 **(p=0.0004)**	0.06 (p=0.4584)	-0.15 (=0.0714)	-0.11 (p=0.1915)	-0.24 **(p=0.0013)**	-0.21 **(p=0.0065)**
GSE14520	0.22 **(p=0.0011)**	0.04 (p=0.5869)	-0.04 (p=0.5669)	0.05 (p=0.4641)	-0.09 (p=0.2031)	-0.19 **(p=0.0043)**	-0.46 **(p<0.0001)**	-0.31 **(p<0.0001)**	-0.20 **(p=0.0031)**
GSE36376	0.14 **(p=0.0255)**	0.06 (p=0.3609)	-0.03 (p=0.6152)	0.02 (p=0.7901)	-0.24 **(p=0.0002)**	-0.26 **(p<0.0001)**	-0.55 **(p<0.0001)**	-0.31 **(p<0.0001)**	-0.47 **(p<0.0001)**
GSE63898	0.13 (p=0.0564)	0.10 (p=0.1394)	0.05 (p=0.4511)	-0.16 **(p=0.0168)**	0.08 (p=0.2452)	0.03 (p=0.6055)	0.01 (p=0.8757)	-0.12 (p=0.0664)	-0.24 **(p=0.0002)**
GSE76297	-0.09 (p=0.2207)	0.15 (p=0.0668)	0.41 **(p<0.0001)**	-0.38 **(p<0.0001)**	-0.14 (p=0.2785	-0.16 (p=0.2207)	-0.34 **(p=0.0071)**	-0.58 **(p<0.0001)**	-0.51 **(p<0.0001)**
GSE5975	0.35 **(p<0.0001)**	-0.18 **(p=0.0048)**	-0.10 (p=0.1397)	-0.18 **(p=0.0045)**	-0.27 **(p<0.0001)**	-0.12 (p=0.0664)	-0.24 **(p<0.0001)**	-0.26 **(p<0.0001)**	-0.25 **(p<0.0001)**
GSE20238	0.06 (p=0.4571)	0.10 (p=0.2584)	-0.01 (p=0.8855)	-0.04 (p=0.6057)	-0.26 **(p=0.0022)**	-0.35 **(p<0.0001)**	-0.45 **(p<0.0001)**	-0.14 (p=0.1151)	-0.22 **(p=0.0106)**
GSE1898	0.08 (p=0.2550)	0.04 (p=0.5829)	0.04 (p=0.6383)	-0.15 **(p=0.0448)**	0.01 (p=0.9151)	-0.14 (p=0.0616)	0.02 (p=0.7995)	0.14 (p=0.0547)	0.02 (p=0.8143)
GSE76427	0.05 (p=0.5996)	-0.02 (p=0.8538)	0.07 (p=0.8538)	0.13 (p=0.1543)	-0.31 **(p=0.0007)**	-0.16 (p=0.0918)	-0.47 **(p<0.0001)**	-0.09 (p=0.8538)	-0.17 (p=0.8538)

S. Table 4. Primers used in this study

Genes	Direction	Primer sequence
GAPDH	F	GTGGTCTCCTCTGACTTCAAC
	R	ATTCGTTGTCATACCAGGAAATG
CAR	F	TGGCATGAGGAAAGACATGATAC
	R	GATCAGCTCTTCTTGCTCCTTAC
CYP2A6	F	TTTTGGTGGCCTTGCTGGT
	R	GGAGTTGTACATCTGCTCTGTGTTCA
CYP2B6	F	AAGCGGATTTGTCTTGGTGAA
	R	TGGAGGATGGTGGTGAAGAAG
UGT1A1	F	GGTGACTGTCCAGGACCTAT
	R	TAGTGGATTTTGGTGAAGGCAGTT
CD24	F	GCACTGCTCCTACCCACGCAGATTT
	R	GCCTTGGTGGTGGCATTAGTTGGGT
CD44	F	CATAGAAGGGCACGTGGTGAT
	R	ATACTGGGAGGTGTTGGATGTGA
CD133	F	CACTACCAAGGACAAGGCGTTC
	R	CAACGCCTCTTTGGTCTCCTTG
EpCAM	F	AATCGTCAATGCCAGTGTACTT
	R	TCTCATCGCAGTCAGGATCATAA

S. Table 5. Reagents for Western blot used in this study

Antibody/Reagent	Source	Lot Number	Dilution
Anti-Constitutive androstane receptor antibody	ab186869, Abcam	GR186869-6	1:500
Recombinant Anti-Vinculin antibody [EPR8185]	ab129002, Abcam	GR3395452-5	1:20000
Goat Anti-Rabbit IgG H&L (HRP)	ab6721, Abcam	017M4850V	1:10000
PageRuler™ Prestained Protein Ladder, 10-180 kDa	26617, Thermo Fisher	01194448	

Supplementary Figures

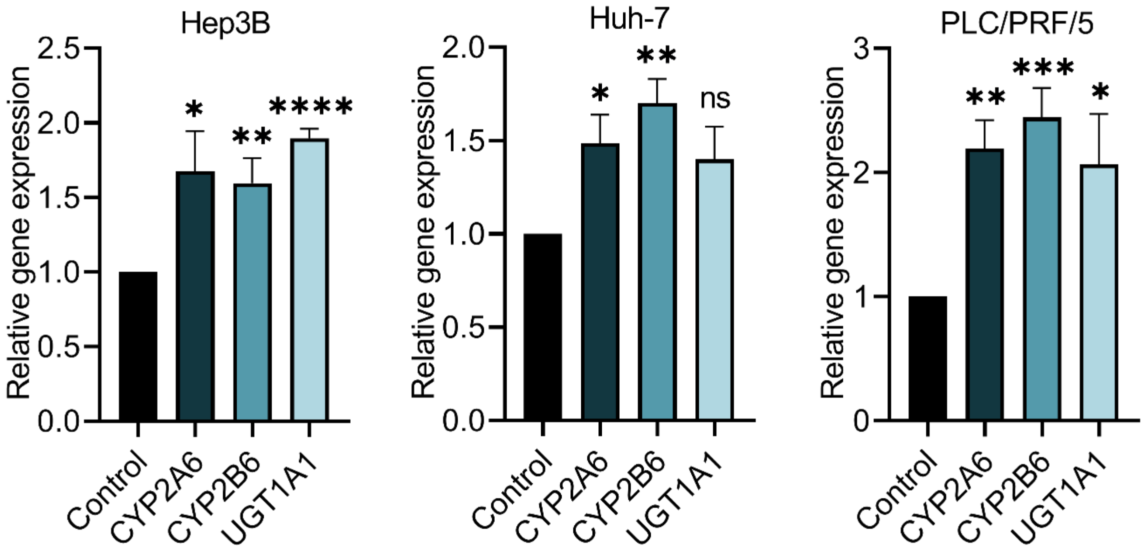

S. Figure 1. Successful CAR activation in HCC cells. (A) HCC cells were treated with 1μM of CITCO. The expression of the downstream targets was examined 48 h later at the mRNA (n=3) by qPCR. Control: Vehicle control; *: $p<0.05$; **: $p<0.01$; ***: $p<0.001$; ****: $p<0.0001$; ns: not significant.

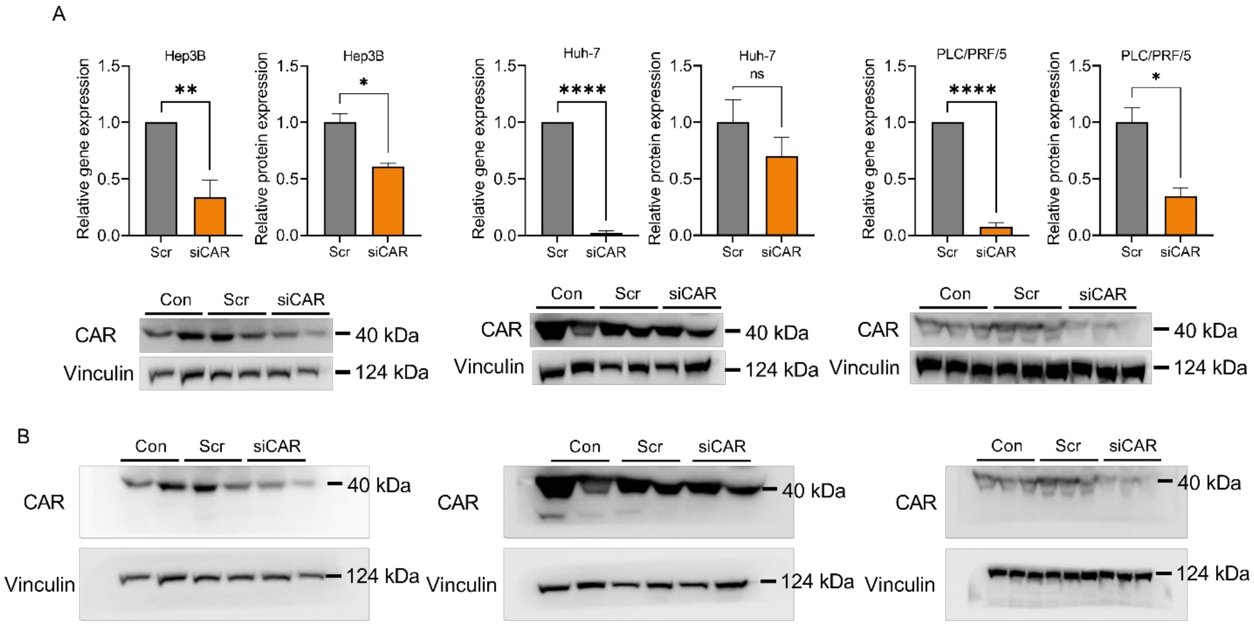

S. Figure 2. Successful CAR knockdown in HCC cells. (A) HCC cells were transiently transfected with 40 nM of CAR siRNA (siCAR). The expression of CAR was examined 48 and 72 h later at the mRNA (n=3 for all) and protein levels (n=2 for Hep3B and Huh-7 and n=3 for PLC/PRF/5) by qPCR and Western blots (cropped blots from the same gel), respectively. **(B)** Un-Cropped images of original western blots shown above. siCAR: cells transfected with CAR siRNA; Scr: cells transfected with scrambled siRNAs. Control: naïve cells. *: *p*<0.05; **: *p*<0.01; ****: *p*<0.0001; ns: not significant.

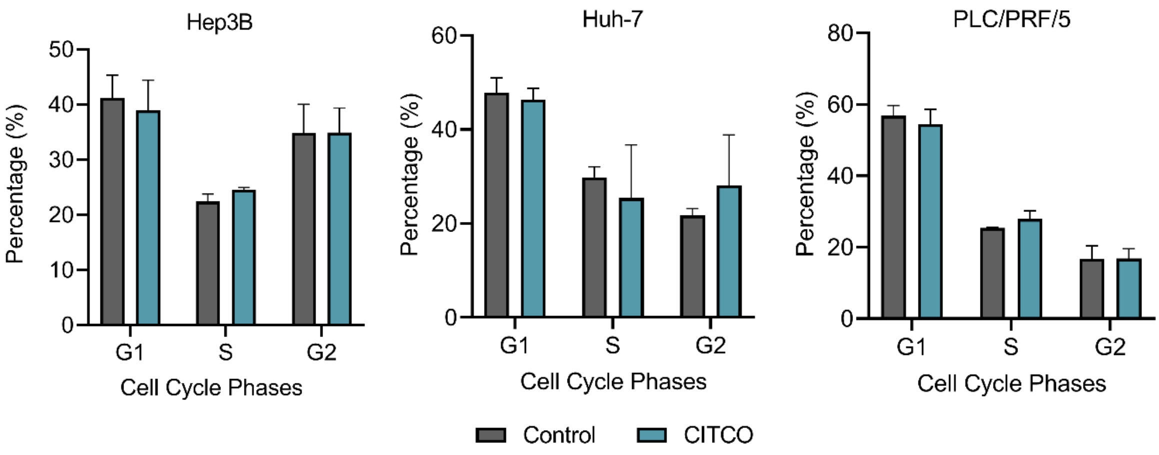

S. Figure 3. Cell cycle analysis in HCC cells treated with CITCO. CITCO-treated cells were analysed with flow cytometry. A decreasing trend in the percentage of cells in G1 phase and a parallel increase in the percentage of cells in S phase can be seen. Data were derived from 3 separate experiments each with 3 replicates.

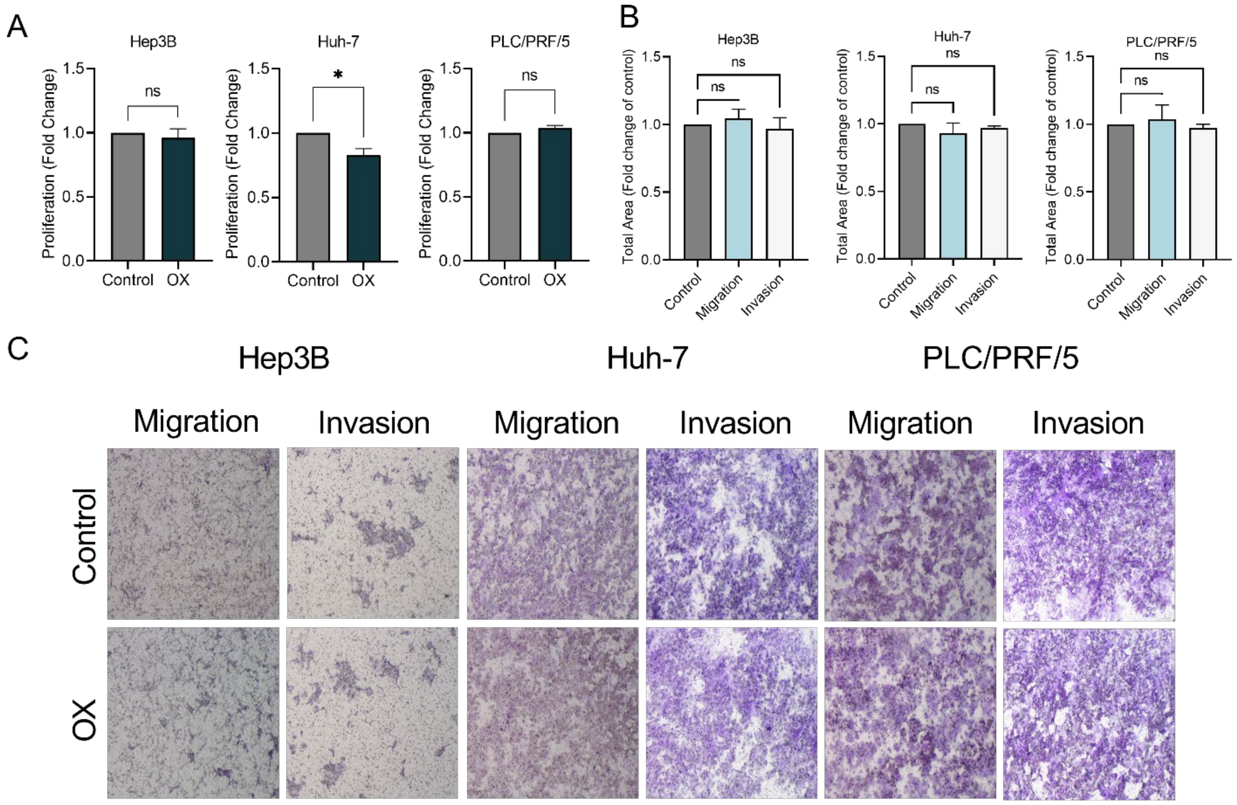

S. Figure 4. Effect of CAR over-expression on the proliferation, migration, and invasion of HCC cells. HCC cells were transiently transfected with CAR over-expressing plasmid. After 48 h of transfection, cells were assayed for proliferation (**A**), migration and invasion (**B, C**). CAR overexpression only mildly inhibited cell proliferation of Huh-7 cells but not other cells (**A**). Transient over-expression of CAR showed no effects on the migration and invasion of all 3 HCC cell lines (**B, C**). Representative images of migration and invasion studies are shown. OX: over-expression. *: $p<0.05$; ns: not significant.

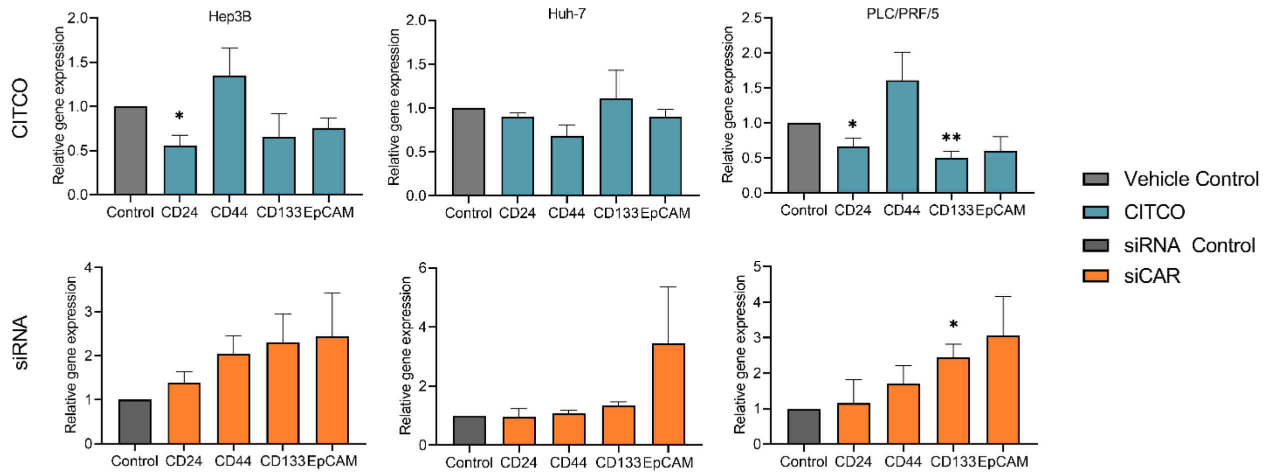

S. Figure 5. Effects of CAR modulation on the expression of cancer stem cell markers. A. Effect of CAR activation by CITCO on the expression of cancer stem cell markers in HCC cells. **B**. Effect of CAR knockdown by siCAR on the expression of cancer stem cell markers in HCC cells. Expression of these markers was examined by qPCR. TS, tumour spheres. *: *p*<0.05; **: *p*<0.01; ***: *p*<0.001.
